# *The time is out of joint*. Teacher subjectivity during COVID-19

**DOI:** 10.1007/s10857-021-09506-3

**Published:** 2021-07-12

**Authors:** Alessandro Ramploud, Silvia Funghi, Maria Mellone

**Affiliations:** 1 Department of Mathematics, University of Pisa, Largo Bruno Pontecorvo 5, 56127 Pisa, Italy; 2 Department of Mathematics, University Federico II, Via Cintia, Monte S. Angelo, 80126 Napoli, Italy

**Keywords:** Teacher’s subjectivity, Crisis, Teacher education, Lacan

## Abstract

In this study, we address the issue of mathematics teachers' personal and professional responsiveness to changing circumstances, such as the shift in external demands made on teacher practice due to the COVID-19 pandemic. For investigating a such delicate issue, we take a theoretical approach, which is quite novel in the field of mathematics education: Lacan's psychoanalytical lens. Specifically, we will use this psychoanalytical lens to analyze a case study focusing on a primary school teacher during the first lockdown in Italy, during which school was organized exclusively in the form of distance education. The analysis of the teacher’s crisis and the strategies she adopted to overcome this crisis give some suggestions about possible directions and issues to consider for future mathematics teacher training proposals.

## Introduction

*Hamlet's* exclamation "*The time is out of joint**"* in this historical time resonates quite strongly with a common sense of time being shattered, out of order. Indeed, the citation is of a scene from which we would like to trace out an analogy with many teachers' sense of self today.

The beginning of the COVID-19 pandemic was a big challenge for schools and education institutions all over the world (Bakker et al., 2021; Engelbrecht et al., 2020a, 2020b). Teaching within such a new context constitutes a real problematic issue for teachers, in our perspective. Indeed, teachers perceive themselves, first of all, as individuals in the context of the pandemic, but they also play the role of teachers, who have a series of educational responsibilities in a context where their relationships – and consequently the vital space of every educational and didactic process – has to be completely re-conceived.

March 8th, 2020, the Italian Prime Minister declared a state of national emergency due to the COVID-19 pandemic and closed all businesses that were not indispensable. In this context, schools also underwent a complete closure. Italian teachers suddenly found themselves in a situation where didactic practices in presence were completely suspended. In the first phase of lockdown, the Minister for Education provided only generic indications about the continuation of didactic activities during the emergency situation, leaving teachers in a sort of “legislative void”. Only after some weeks of lockdown did the Minister for Education begin to talk to the press about the possibility/necessity for teachers to engage in DE.

Meanwhile, research on DE in Mathematics Education was not so developed as to provide Italian teachers with research frameworks to rely on for the redefinition of their whole didactic practice in a context in which they could *exclusively* recur to remote communication. This situation generated various reactions from the teachers – for example, many created tutorials about technological instruments, others shared didactic practices reread in light of DE, others yet refused to engage in didactic activities during the lockdown, contrasting DE on the basis of the fact that it was not something they were expected to do based on their National Contract.

All these elements generated, as it could be easily guessed, a sort of rupture in the consolidated teaching habits teachers had before the COVID-19 pandemic. From this point of view, we can say that for teachers, the lockdown revealed to be a real moment of crisis. In order to characterize this crisis situation within the horizon we will try to set in this paper, we believe it is interesting to consider the description of crisis situations by Yerushalmi (2007):

*Crisis situations, as I am defining them, occur during ordinary life occurrences when one's personal mechanisms for organizing experience cease to function. One's once familiar procedures for responding to and acting on the environment are disrupted or obliterated. This follows upon powerful emotional processes, which lead to the reversal or destruction of mental patterns or schemas which had been created through repeated experiences of self-versus-other and which had left memory traces in the psyche. [...] Often the primary experience is one of internal imbalance and lack of control over one's life.* (pp. 359–360)

In relation with this kind of characterization of crisis situations, we also find very interesting Yerushalmi’s description of the process of overcoming crisis situations by means of a new organization of the self («an emergence from crisis always involves the development of a new and different organization of the self», ibidem, p. 360). In the attempt to describe the possible different ways to overcome the crisis situation in which Italian teachers found themselves, our choice was then to study this process of reorganization of the self, starting from the construct of *subjectivity*.

## The Lacanian lens of subjectivity to interpret mathematics teacher’s crisis situation

Within the previous literature about subjectivity in Mathematics Education (e.g., Chronaki & Pechtelidis, 2012; Dowling, 1991; Evans & Tsatsaroni, 1994; Palmer, 2009), we find particularly interesting Brown’s approach (). According to him, Lacan «provides a bridge from mathematics education research to contemporary theories of subjectivity more prevalent in the cultural sciences» (Brown, 2008, p. 1). Subjectivity in this perspective is no longer seen as a unified entity, but it becomes a construct where continuous internal tensions take place (see Lacan, 1988). Brown in some of his previous studies (2011, 2014) employed a Lacanian perspective to analyze the relationship between the subjectivity of the individual, on the one hand, and the governmental regulation and university systematic knowledge, on the other hand. Therefore, the “legislative void” and the lack of theoretical frameworks developed to realize teaching *exclusively* remotely constitute an unprecedented situation to be studied taking a Lacanian perspective on subjectivity.

The analytical model introduced by Brown (2008, 2011) consists of a multidimensional model. He describes subjectivity in this way:

*Lacan’s notion of subject is based on three orders: the Imaginary, the Symbolic and the Real [...] the Imaginary might be seen as self-identification, or rather, the creation of images of oneself [...] The image of self, as characterized by a name, fixes an egocentric image of the world shaped around that image of self. [...] While the Imaginary might be seen as the individual looking in on a fantasy self, the Symbolic encapsulates this individual looking out to a fantasy world filtered through the ideological framings brought to it. [...] The Real might be seen as the space in which the Imaginary and Symbolic are enacted. The fantasies built within the Imaginary and the Symbolic fail to capture, respectively, the signified self and the signified world. This brings into play a space for desire motivated by the supposed possibility of closing the gaps between the supposed Imaginary and Symbolic and the Real that hosts these dual fantasies. The Real, by definition resists symbolisation. The resources of language cannot mop up the whole of experience.* (Brown, 2008, pp. 237–238)

It is necessary to specify that in the Lacanian perspective, these three orders are bound to each other by a mutual connection, in which each order affects the others but none of them is privileged (Brown, 2011). Nevertheless, for what concerns our following discussion, we need to emphasize the relation between the Symbolic order and the other two orders.

*Lacan’s conception of society is dominated by the practice or use of language, where ‘when I say use of language I do not mean we use it – it is language that uses us’* (Brown, 2014, p. 285)

Since the child gets into a linguistic-cultural-communicative dimension, on the one hand, such dimension determines the cultural filters through which s/he will shape her/his own *Imaginary* («The Symbolic provides a yardstick against which I can measure myself and understand myself in relation to the social frame. That is, the self is not egocentric but defined in response to social expectations», Brown, 2011, p. 124), and, on the other hand, it gives life to the *Real* order through its own breakings – i.e., what the linguistic-cultural-communicative dimension cannot express or signify («for Lacan the Real […] is not an external thing that resists being caught in the symbolic network, but the fissure within the symbolic network itself», Žižek, 2007, p. 72). From this point of view, the interaction between the three orders becomes inextricable and always corresponding to a codetermination.

Given this, we would like to try to read Italian teachers’ crisis situation taking a Lacanian perspective: it may not be easy, in fact, for some teachers to reread elements of the *Imaginary* (one's fantasy vision of self as a unitary person, as a teacher) and of the *Symbolic* (the socially and culturally shared construction of who is a teacher, what it means to teach, etc.) in order to fit into the new aspects reality presents nowadays. Therefore, we believe it may be useful to introduce this characterization of subjectivity in order to study teachers’ processes of adaptation to crisis situations.

Some studies in Mathematics Education have already highlighted the need to study the way people manage crisis situations—for example, Antognazza, Di Martino, Pellandini & Sbaragli (2016); Di Martino & Gregorio (2019). In our case, the change in schools' functioning introduced by the contextual conditions due to the COVID-19 emergency is not only a real problem for teachers to solve, but it can also be seen in terms of a profound changing of the whole cultural system in which teachers live and work (Engelbrecht et al., 2020a, 2020b).

In our framework, subjectivity is deeply linked to the cultural context (Brown, 2017): «Subjectivity […] relates to individuals whose psychological existence is distributed across a multitude of linguistic filters» (Brown, 2011, p. 90); «Semiotic systems are culture dependent and subjectivity is entwined in each dimension of this dependency and what you see results from this entanglement» (Brown, 2011, p. 129). So, if we take this perspective, the cultural change teachers are facing can be put in parallel with one of the basic assumptions of the Cultural transposition (CT) framework for teachers’ professional development. CT is a perspective in which cultural aspects are crucial, and it is aimed at giving teachers, who have come into contact with teaching practices from different countries, the opportunity to become aware of their own unthoughts, i.e., some of the “invisible” cultural beliefs about teaching/learning they have absorbed by their own culture (Bartolini Bussi & Funghi, 2019).

## The cultural transposition perspective to connect the reality of COVID-19 pandemic to the Italian context

Recently, the perspective of Cultural transposition (CT) was proposed to frame and design the encounter of teachers with Mathematics Education tools and methodologies coming from different cultural contexts (Mellone et al., 2019, 2020). For example, it was used to frame the encounter of a group of Italian in-service primary teachers with the “problems with variations” method, an educational tool used in Chinese primary school that can foster early algebraic thinking. This encounter, at first, lead Italian teachers to face a moment of crisis and, later, thanks to lead of educators, to realize the lack of attention towards algebraic thinking in the Italian primary school practice (Mellone et al., 2019). These encounters between teachers and teaching practices from foreign countries, and the connected experiences of crisis, are proposed during professional development courses designed for supporting the participating teachers in developing awareness about their teaching practice and in emancipating themselves in choosing appropriate educational tools based on their educational intentionality. Indeed, in our perspective, the experience of emancipation potentially offered to teachers by professional development courses framed in CT should help them to develop skills connected to specific mathematics topics (for example, algebra), but also more transversal skills, that we can call *coping skills*, such as finding one's educational intentionality and flexibly trying to find ways to design one's educational proposals corresponding to such an intentionality, even in unexpected situations – we can also speak of *resilience skills*. This, of course, does not mean to try and avoid teachers experiencing difficulties, but rather to offer them tools to face and overcome frustrating feelings and difficulties.

Unlike with the previously mentioned CT experiences, the cultural change teachers have faced during the COVID-19 emergency has not been a training experience proposed, controlled and accompanied by any group of researchers. Rather, it was a violent and uncontrolled cultural change that teachers had to face at a moment of extreme isolation, since communication with and among students was to be necessarily combined with technological tools, changing the nature of communication (Engelbrecht et al., 2020b)—so that researchers have raised the question regarding what kind of teachers’ professional development is needed in this new context and how professional development courses should be designed (Bakker et al., 2021). Despite the fact that CT experiences and the COVID-19 pandemic are based on different assumptions, the crises we observed in Italian teachers in this emergency time has sent us back to the crisis experiences which is the main assumption of the training process through CT. For this reason, we identified a framework to help us read teachers’ crises in relation to subjectivity with a new lens. Brown’s approach (2011) seemed particularly interesting to us, because it refers to Lacan, a psychoanalyst who describes the whole development of subjectivity in terms of *alienation* – i.e., the process of understanding one’s self as something different from everything outside.

In order to better explain the approach we will use to analyze our case study, we still need to outline some of the fundamental elements that affect the Italian cultural context.

In Italy, the general cultural context and, in particular, school culture, are still affected by the historical events that took place at the beginning of the last century and, in particular, by the school reform dating back to those years. Those years are characterized by the end of the First World War and the beginning of fascism in Italy; the protagonists of the school reform are, on the one hand, the great mathematician Federigo Enriques—a strong supporter of the centrality of the exact sciences for the technological and more widely cultural development of the country—and, on the other hand, Benedetto Croce and Giovanni Gentile—who tended, instead, to limit the weight of mathematics in the cultural landscape of the country. The school reform, however, was signed, in the end, by Gentile, and not by Enriques, with the consequent downgrading of the exact sciences in the cultural development of the country.

These historical events supported a humanistic perspective on knowledge, at the expense of a good development of the relationship with mathematical-scientific-technological knowledge. In addition to this historical element, there also was a delay in infrastructural development of the educational environment (for example, the first infrastructural projects aimed at making the connection cables reach the schools date back to around twenty years ago) – due also to a geographic conformation of the Italian territory, which constituted an obstacle to the physical building of Internet connection networks.

In an attempt to briefly outline some of the problematic issues Italian schools had to face during the lockdown, we can identify three main points:

1. From an historical viewpoint, there was a lack of development of teachers’ mathematical-scientific-technological abilities rooted in the history of Italian school system and Italian teacher education in the first half of the 20^th^ Century;
2. From a geographic viewpoint, as we have just highlighted, there are many problems at an infrastructural level regarding the spread of informatic technologies;
3. Finally, on the level of teachers’ professional development, these aspects have supported attitudes that can be described as a “technological refusal”.

Another important issue is the fact that in Italy *freedom of teaching* is a fundamental right, which since 1948, has been enshrined within the Constitution. The teacher has no strict guidelines, but, rather, national guidelines (called National Indications) that do not express specific teaching obligations, but that trace out broad goals to be reached by specific years. The presence of the freedom of teaching as a right included within the Constitution is due to the attempt—in times of post-Fascism—to defend teaching from authoritarian deviations of democracy.

These issues got entangled with the situation generated by the lockdown and school closure in a messy way. On the one hand, teachers’ freedom of teaching guaranteed by the Italian Constitution and the technological infrastructural problems made it substantially impossible to impose guidelines on DE that were valid all over the national territory; on the other hand, Italian teachers’ scarce technological skills made them strongly unprepared to face the difficulties of a way of teaching relying exclusively on remote communication. Nevertheless, this fading away of teaching in presence—typical of the Italian cultural school context—and the new didactic opportunities provided by technological platforms to be explored, allowed the teachers with more advanced technological skills to experiment new ways of teaching, and therefore also to re-think their didactic practice.

In this perspective, we trace out a parallelism between the situation teachers faced during COVID-19 pandemic and the situation that is created through professional development courses based on CT. According to Lacan (2006), we observe that the “discourse of the Other” is not only the cultural context within which the individual grows and is educated, but it is also the base on which subjectivity is shaped: whereas in CT the teacher is driven towards a different “discourse of the Other” (because s/he is exposed to didactic practices coming from foreign cultures, which therefore refer to symbolic elements that are different from those of her/his own culture), in the situation generated by the lockdown the “discourse of the Other” remained substantially unchanged, albeit making it practically impossible to realize the didactic practices (in other words, the pedagogic and didactic references remained the same, but it became impossible to follow them because they are based on the sharing of the same physical space by the teacher and the student).

However, both situations can be seen as contexts in which the teachers have to reflect on their usual pedagogical and didactical premises, beliefs and aims—those elements that in the CT framework are summed up with the word “un-thought”, because they are taken for granted at such a high level in the usual didactic practice that they become “invisible” to the teacher her/himself. The crises generated by the two situations (COVID-19 lockdown and professional development courses based on CT framework) in teachers’ didactic practice—intended as moments of disruption of teachers’ way of acting within the school environment – seem particularly interesting to investigate in order to understand which differences and which similarities there could be in the reflection of their own “un-thoughts” generated in the two contexts, in the hope that this could also shed a new light onto the strategies enacted by teachers to overcome their crises in both cases.

## Research question

In order to help the reader understand how the present study is nested in the theoretical and contextual frame we have traced out, we introduce two research questions—that for us work as a compass—guiding our research interest in the horizon of the problematic context due to the COVID-19 pandemic.

1. Can teacher trainings framed in the CT perspective support teachers in the management of crisis situations?
2. In particular, is it possible that the continuous exposure to crisis situations created by these CT teacher trainings helps the teachers become more flexible in the continuous rethinking of their teaching activity?

In the present paper, we will focus in particular on the following research question:

- Which needs and suggestions for teacher training emerge by analyzing the interviews of an expert teacher during her experience in COVID-19 time, through the three orders of Lacan's subjectivity?

## Methodology

During the 2019–20 academic year, the first two authors had the opportunity to monitor teachers from various Italian regions, within the PerContare project (www.percontare.it), with monthly meetings and frequent exchanges of teaching material and consultancy for lesson planning. With the goal to partially face our research question, we chose one teacher (that we will call Sophia) for the case study. Sophia was an experienced and highly trained teacher. Sophia has been teaching mathematics for about 15 years, and she was very well trained in Mathematics Education, in particular in studies carried out by Italian research groups. She has participated for many years in professional development courses and experimentations with the Mathematics Education research group of the University of Modena and Reggio Emilia. Sophia was very experienced in Semiotic Mediation and Mathematical Discussion (Bartolini Bussi & Mariotti, 2008), and in particular in the CT of Chinese Lesson Study (Bartolini Bussi et al., 2017) – didactic approaches that she used in a widespread way for planning lessons and for didactic management. We will refer to these didactic approaches in the following using the expression “semiotic mediation approach”. She also used in a massive and varied way "mathematical problems" as a teaching tool, in various forms (for example, narrative problems, Chinese problems with variation, problems from standardized tests, etc.).

Sophia’s solid didactic preparation made it interesting for us to trace out the difficulties she met and the strategies she found as she faced the new contextual conditions – which, as we will see, were not very consistent with her view of school and of teaching. Moreover, her deep knowledge of Lesson Study and CT made it also interesting for us to investigate whether her previous experience with CT was having some kind of influence on her current way of managing the new contextual conditions. In addition, we chose to analyze this case because of Sophia’s reactivity to the lockdown and to the new constraints imposed on her teaching by DE. Although Sophia showed difficulties and a personal crisis in her profession during the lockdown – as we will see in the following – she continuously attempted to find teaching solutions, sometimes drawn from her educational background, so that she could continue to engage in teaching–learning processes with her students. She was also very well prepared from a technical viewpoint – she was one of the referents for technology in her school. Her ability in this matter distinguished her from other teachers in our eyes, because the main difficulties we detected in other teachers were mostly due to their lack of familiarity with technologies, so we found Sophia’s case interesting to raise issues that go beyond a lack of technological ability.

We chose to conduct a case study, mainly through the collection of narrative material. We collected two semi-structured interviews, lasting 1 h and 30 min each, one on April 1^st^, 2020, and one on May 1st, 2020, focused on the following topics: changes that happened during the lockdown, both in teaching practices and in teacher self-perception; moments of personal crisis and difficulties, and strategies used to overcome them; kinds of professional development resources consulted during the lockdown. We chose to conduct two interviews one month apart to trace out changes that occurred during the month of April. Both the interviews have been audio-recorded and fully transcribed. In addition to this material, the first two authors collected field notes throughout the academic year, which have been used to complete the data collected through the interviews.

*Rationale* «Subjectivity locates the individual, in relation to the stories they tell of themselves, the stories they tell of the world, and the stories people tell about them. The individual is not so much seen as a cognitive or biological entity» (Brown, 2011, p. 84). In these words, we can see subjectivity as strictly connected with a storytelling process: we deemed it appropriate to study Sophia’s *Imaginary*, *Symbolic*, and the way she thinks to enact these two orders in the *Real* through a qualitative analysis of narrative material – as it has been already done by Brown (2011), who analyses many cases starting by material collected through interviews or teachers’ diaries.

Nevertheless, «the choice of the research instrument is never neutral» (Di Martino & Funghi, 2016, p. 213). We chose the interview as research instrument because we believe it to be more suitable with respect to other research instruments – as questionnaires – to gather in-depth information on Sophia’s perception of herself and her relationship with the crisis context. The choice of the semi-structured interview was due to its greater flexibility with respect to the structured interview (Cohen, Manion & Morrison, 2007): this flexibility made it a suitable instrument to investigate relevant issues for Sophia as they emerged during the conversation.

The data were initially read over and over in order to identify by large Sophia’s point of view (Patton, 1990). We then passed to data coding, taking a bottom-up approach (Rieman, 1998). We underlined the main emergent themes, paying particular attention to the coherence between interviews and collected field notes. After this coding phase, we identified possible recurrent patterns and links among emerged themes (Rieman, 1998). At this point, the first two authors resolved all detected discrepancies in the interpretation of data. This analysis was presented to the third author, in order to check its validity and trustworthiness.

In particular, we looked for patterns indicating the presence of a crisis situation – seen as a fissure in Sophia’s usual way of teaching and in her view of school with respect to the mandatory constraints she had to stick with – and attempts to overcome the situation – seen as attempts to build new ways of coping with the current environment. As textual indicators of a crisis situation, we looked for references to emotions, such as anxiety, uncertainty and frustration, and for verbs as “put into crisis”, “miss”, “do not know”; for the process of overcoming the crisis situation, we looked for words expressing emotions such as happiness, relaxing or relief, or verbs as “overcome”, “return”, “reinvent”, “recreate”. This choice was inspired by previous studies that have emphasized the importance of the capability to manage negative emotions such as frustration and shame to overcome crisis situations (Antognazza, Di Martino, Pellandini & Sbaragli, 2016; Di Martino & Gregorio, 2019). Then, we reread this analysis on the basis of Brown's framework on subjectivity, identifying aspects related to the perception of oneself and one's image as a teacher (*Imaginary* order), aspects related to one's personal beliefs about school and teaching (*Symbolic* order), and aspects referring, instead, to contingent and contextual elements. Since the *Real* cannot be covered by the *Symbolic*, which also includes the language, it is not possible to trace elements of Sophia’s *Real* order starting from her words, so we did not look for it in the transcriptions. We also looked for textual indicators in Sophia’s words to describe her view of herself (for example personal pronouns as “myself” or expression as “my perception of my role”, “As a teacher, I…”), her view of school and of teaching (for example expressions as “for me school is…”, “the school I imagine…”, “the role of teacher is…”), and words hinting at contextual factors (for example expressions describing unedited conditions, as “a new way of staying with children”, “something nobody has ever experienced”, “something we were not prepared to face”).

## Data analysis

### ***First interview, April 1***st***, 2020***

#### Crisis of Sophia’s perspective on mathematics teaching

In the first month of lockdown, Sophia describes a great difficulty to understand how to operate in the new context: how to recreate the network of contacts with students, provide them with devices, explain and train families to use technologies, understand which platforms to use and how to teach with them. The sudden interruption of teaching activities in presence introduces an element of *alienation*, compared to the previous normal situation.

Sophia: At the beginning — namely, the first weeks, I would say also the first month — it was a very difficult moment for sure [...] that phase was really a complex phase, which certainly put me in crisis because I didn't know what would happen to my two classes.

We can read this crisis in the words Sophia chooses to describe her situation: “*it was a very difficult moment for sure”, “a complex phase, which certainly put me in crisis”.*

Sophia: At the beginning, my perception of myself as a teacher was very different: it was closer to an ‘educatore’ [closest English translation "educator"], and therefore I was more attentive to the relational aspect. That is, in the first and second week of lockdown, my urgency was to reach students, I mean: to keep in touch with students, but in order to restore an affective relationship rather than a professional contact.

Sophia’s perception of an interaction that has become more cumbersome (both among pupils, and between teacher and students) was accompanied by a sort of shift in the perception of her role: she felt more as if she was an “*educatore*”^1^ than a teacher, because she privileged the care for relationship with respect to curricular contents. In Italy, “*educatore*” is a professional figure that is different from the teacher, because his/her role is aimed to reduce the gap between students with special needs and the others; an “*educatore”* is not a discipline-expert but an expert in taking care of the human relationship with students. This initial shift we can read in Sophia’s words indicates a temporary disruption in her *Imaginary* order (*“my perception of myself as a teacher was very different*”, *“my urgency was…”*)*,* since it denotes a change in Sophia’s perception of herself—i.e., her mirror image—as a teacher in reaction to contextual and contingent needs. The use of the expression *“my perception of myself […] was closer to an ‘educatore’”* testifies to how in Sophia’s *Symbolic* the responsibilities of teacher and of the *‘educatore’* are clearly separated: in this perspective, the shift towards a greater care of her relationship with the students represents a great change in Sophia’s *Imaginary*, because it is something close to “putting herself in the shoes” of a different professional figure.

After this first moment, Sophia describes in DE what we could call a crisis of her "semiotic mediation approach” of Mathematics teaching.

Sophia: What I miss most of the class is the whole aspect of sharing thoughts, strategies, even of mutual correction among children in the moments of mutual exchange, of argumentation; it is a very important moment when they manage to listen, manage to interact and converse with each other, pursuing their own ideas and points of view.

Talking about the constraints of the exclusive use of DE, Sophia emphasizes a lack of moments for interaction and discussion between pupils, and the absence of ways to simulate teamwork or cooperative learning and to orchestrate mathematical discussions.

Sophia: While working in the classroom you are able to grasp a number of gestures, movements, signs, interaction between children, which allow you to work with them more effectively […] This relationship between children, even physical *[i.e., gestures, movements, signs, etc.]*, is missing in DE, while in the school I imagine, the school where I work, I try to value all these gestures, all these activities of mutual exchange.

Sophia explains that in DE she misses pupils' gestural language and proxemics, and this makes more difficult for her to understand pupils’ needs.

This view of school is recurrent in Sophia’s considerations. Indeed, the current DE experience is not perceived as a real school, but rather as a fragmented experience, that teachers were absolutely not prepared to face; this “school” is seen as something teachers are inventing on the spot. Sophia summarizes this by saying that the school *“is relationship, it is interaction, and therefore what we are experiencing is too far from these things”* (to be “real” school). The feeling of missing “real” school can be read in light of our framework as a sign of an enhancement of the imbalance between Sophia’s *Symbolic* order and the reality she has to face – i.e., between her view of school (*“in the school I imagine…”*) and the constraints of DE.

In the first interview, anxiety emerges as an emotion characterizing the preparation of the first lessons, due to Sophia’s difficulty to anticipate the lesson progress.

Sophia: I still don't really know what will happen in the lesson I prepare, so I found myself having to manage a little more anxiety.

In addition, Sophia underlines some issues related specifically to her experience with live lessons in streaming, as the issue of very short times.

Sophia: Times are very short. If I am at school, I have two or three hours to spend with the children; here I have a maximum of half an hour, and anyway it's a lot because I see that after fifteen, twenty minutes that I work with them they are very tired... and so I really have to keep very tight times. Both these facts cause difficulties in finding the time to write down some formalization. In my opinion, formalization is much more urgent in this modality compared to work at school, because at school [...] there are many moments in which we take the time to think about what we understood. In DE no: I miss all these moments, because I have very few moments in which I meet children.

She highlights the problem of very short lessons and limited attention from students – we note that during the lockdown Sophia met her students in live streaming lessons for two periods of 45 min per week on Mathematics, instead of the 7 h per week she was used to spend doing mathematics with them before the school closure. Lack of time to formalize is an element indicating a distance between the current way Sophia is able to teach and her *Symbolic* order, since formalization is one of the fundamental parts of her didactic approach.

### First signs of reorganization of Sophia’s way of teaching

Sophia: In order to be able to overcome this anxiety, I tend to prepare myself better to [respond to] pupils’ possible responses.

In order to react to her emotion of anxiety, Sophia restored a greater emotional balance paying more attention than ever to a priori lesson planning. In fact, even if lesson design is considered important both for teaching in presence and teaching at distance, for Sophia it becomes essential in DE because of the short time available to interact directly with students.

Sophia: Lesson planning is fundamental, I would say that in DE it is absolutely essential. While in presence teaching it is also possible to design with less detail […] when time is so short, design is an element you cannot renounce to, and it must be done precisely.

We read both anxiety and this “hunger for time” as signs of alienation, of Sophia’s feeling of being “unfit” for the new context – hints of an attempt to enact her *Imaginary*-*Symbolic* orders in a reality that is *out of joint*. Nevertheless, the establishment of a phase of a priori lesson planning before each lesson constitutes one of the first signs of an effort to react to the crisis situation and to rebalance this alienation—indeed, Sophia uses the word “overcome” (“*to be able to overcome this anxiety…*”).

Sophia: [In this experience of DE] I had to rethink my profession, my teaching methods [...] it helped me to look for the real crucial elements [of my way of teaching], I mean... to remove all the "bells and whistles" from the things I wanted to do with the children […] therefore [it helped me] to understand what are the crucial points for me among the huge amount of stuff we have to do at school, and to understand how I could face it with my pupils in a very new, very different way, and how to modify my teaching in a very different context.

This need for careful lesson planning pushed Sophia to identify what she cannot do without, in order to exploit the time at disposal with her students to work on what she thinks is really essential. Especially the expression “*how to modify my teaching in a very different context"* is a clear indication of this search, needed to restore a “dialogue” between her way of teaching – connected with her *Imaginary*-*Symbolic* orders *(“understand how… to modify my teaching”)* – and reality *(“a very different context”)*.

Sophia: I believe that Lesson Study structure was useful to help me “tighten up” the lesson, find the key points I wanted to make children think about; to get used to managing a very short time; to recreating a relationship that even in a short time could include some features of my daily teaching, namely: task proposal, discussion about it, and formalization of the core content.

Sophia highlights that, in this search for what she thinks is essential, a crucial role was played by her CT training on Lesson Study as part of her professional development background: she exploited her expertise on Lesson Study in order to design teaching modalities that were compatible with the timing of DE without losing the focus on the essential curriculum. She sees her Lesson Study training as valuable for setting up lessons in live streaming with students, when the teacher wants to deal with conceptually complex contents, where student–teacher interaction is needed to build meaning with little time available.

Sophia: My training in Lesson Study allowed me to try to deal with complex things in those few minutes of the weekly lesson, things as contents, modalities, way of reasoning that could not have been presented through “aseptic” video-lessons, without interaction [...] I don't think without the preparation of Lesson Study I would have been able to do it, because half an hour is really very short.

In our perspective, CT training on Lesson Study is exploited as a means to enact a compromise between her *Imaginary-Symbolic* orders (“*recreate a relationship that… could include some features of my daily teaching*”) and the new constraints of the reality (“*those few minutes of the weekly lesson*”). The reduction of Mathematics lesson time (from 7 h to two periods of 45 min per week) and the changing of the conditions in which Sophia has to teach are elements that she tries to re-interpret in light of a *Symbolic* dimension which poses children's interaction at the center of teaching–learning process.

In this perspective, Sophia also exploited the possibilities provided by the Italian school cultural context, which is not so prescriptive, to redefine curricular goals. In particular, since she was teacher in the 2nd grade during the lockdown, she chose to focus on multiplications and divisions, as key content to be covered in DE.

#### ***Second interview, May 1***st***, 2020***

### Sophia’s refining of strategies to cope with DE

Between April and May, 2020, Sophia succeeded in identifying some strategies to deal with the lockdown – which we can read as means of re-organizing her way of teaching, searching for a way to balance her *Imaginary* and *Symbolic* orders with DE constraints emphasized in the first interview. Firstly, she tried to create playful and practical activities to get pupils involved – sometimes starting from suggestions collected by other teachers, or through the constant dialogue with her colleagues.

Although active learning is not seen as very compatible with DE, it still remains indispensable for Sophia: manipulative activity is seen as a fundamental part of the learning process, especially for the lower grades; the teaching–learning process is conceived as inextricable from human relationships and discussion.

Sophia: I would say that one of the fundamental aspects of my teaching is the relational one, because you learn together, and therefore I don't want to give it up, in any way. [...] An active teaching-learning is not only made of pure abstraction but of concrete manipulatives, on which you can operate – for example cut, paste, etc. These are all physical actions which are in my opinion an important means of conveying mathematical contents, even abstract ones. Above all with small children.

Therefore, Sophia tried to reproduce her “semiotic mediation approach”, as much as possible, both in activities she proposed in a blended mode and in those proposed during live streaming lessons – an attempt to “close the gap” between her *Imaginary*-*Symbolic* orders and reality. For live streaming lessons Sophia tried to create a format similar to the one described for lessons in presence. In this format, Sophia designs a manipulative activity to be carried out by her pupils, who will experiment it simultaneously, since they are in live streaming with their classmates. Then, Sophia asks each student to show what s/he has produced with his/her experimentation and elicits the discussion and exchange in order to let pupils move toward a shared collective reflection on these productions. For example, to introduce divisions, Sophia proposed to her students an activity in which they had to distribute 24 maccheroni into 2 and 3 plates, and then she elicited a reflection on different distribution strategies asking to her students to explain how they completed the task, and to compare different strategies – see also Sophia’s plan in Appendix.

Sophia: I understood that I have to think about... the time available, and divide it among the tasks, the activities, also - in the last lessons I tried to do it - by simulating the lesson, that is, I imagine and simulate the lesson at home to understand the time needed.

Regarding lesson design, Sophia continuously worked on her way of planning live streaming lessons, in particular on her constant refinement of the lesson plans, aimed to eliminate downtimes and to make the lesson more engaging, consistently with the model of Lesson Study. Sophia also spontaneously added to this planning process a simulation of the lesson to be prepared. We can read this choice as part of Sophia’s way of coping with the difficulty to anticipate the lesson progress, emphasized in the first interview: simulating the lesson indicates an effort to further reduce the gap between her *Symbolic* and reality. This effort is also shown by Sophia's enhancement—for live streaming lesson planning—of some fundamental elements characterizing her presence lessons: the strict selection of a thematic core to be developed within the time available, and the presence of a scheduled moment dedicated exclusively to formalization following the manipulative activity and the related discussion.

Sophia: I try to think of lessons that have a thematic core, a topic - even only a small part of the subject - which, however, has to begin, develop and being formalized [within a lesson], so I try to find the time to formalize, even by making the children write in the lesson one or two sentences useful for summing up.

Broadening to the planning of the academic year curriculum, Sophia’s choice was to make a very stringent selection of the core topics to be addressed in this last months, for example choosing to delay the first approach to Geometry to the next year.

Sophia: Regarding the progress of my annual plan, it is clear that I quickly calmed down since I understood that it would have been not possible to do everything, and I chose the topics to deal with, so I have overcome what could have been a problem – namely, deciding what to teach during these months – by choosing which themes to present and which not.

### Rebalancing Imaginary-Symbolic orders with reality: the importance of “reshaping”

This process of balancing tensions between *Imaginary-Symbolic* orders and the current context is indicated also by the fact that the perception Sophia outlines of herself in the second interview seems to be rather constant throughout the lockdown period.

Sophia: When the interruption of school activities has been prolonged [...] my professional role of teacher returned to be important [...] I recovered what was my profession as an ‘ante-coronavirus’ teacher, therefore I felt again the will to continue my work.

Apart from the first moment, the reflections we collected testify a quick return to a self-perception as a teacher. The recovery of her professional role in this excerpt is indicated by expressions as *“my role… returned”*, *“I recovered… as an ‘ante-coronavirus’ teacher”*. Sophia identifies as an engine of her search for strategies to overcome the crisis situation an intrinsic motivational drive she defines:

Sophia: The desire to reinvent one’s self, to re-imagine a way of staying together with the students, to learn with them, to work together in a new, different way, that nobody had ever experienced.

This excerpt shows in particular Sophia’s view on reaction to alienation generated by the crisis situation: the key is to act at the *Imaginary (“reinvent one’s self”)* and *Symbolic* orders (“*re-imagine a way of staying together with the students”)* in order to cope with an unprecedented reality (*“a new, different way, that nobody had ever experienced”)*.

In Sophia’s case, we can see that her way of teaching in DE consists in a sort of compromise between a selection of the elements at the basis of her *Imaginary* and *Symbolic* orders and the constraints she was obligated to act within. In the *Imaginary* order, the fact that she succeeded in recovering the relationship with her students without losing the focus on mathematical contents allowed Sophia to return to recognizing herself in the role of Mathematics teacher again – even if she lost the physical contact with her students and the possibility to see most of their gestures. In the *Symbolic* order, Sophia actually shows that she succeeded in creating lessons that simulate the dynamics she had in her previous way of teaching, even if she lost her habit to use teamwork on manipulatives for her students. If we assume that *Imaginary* and *Symbolic* orders determine how Sophia acts in the new environment, we can see that Sophia’s subjectivity is altered to let her overcome temporarily the situation of lockdown.

Sophia: Testing my capability to react – and I'm happy that my team colleagues and I took the challenge, and we were able to react to it – and so understanding this is a professional enrichment.

Sophia’s feeling of happiness about her ability to react is a further element signaling her perception of success in coping with the crisis situation – something connected to the coping and resilience skills previously mentioned, that we can interpret in our framework as management and realignment of some elements of *Imaginary*-*Symbolic* orders to reality*.* More in general, Sophia recognizes a “reshaping” capability – namely, the ability of exploiting expertise acquired in one’s own professional development background to adapt to the needs of the moment – to be crucial to overcome this moment of difficulty:

Sophia: years of school experience in my opinion teach to be less rigid, but to be much more flexible and able to adapt to different situations. […] in this DE the constant and continuous exercise of ‘reshaping’ has helped me to not be frightened and to always look for the positive aspects that this situation could carry.

The importance of this aspect is indicated by the fact that Sophia explicitly describes “reshaping” as a way to cope with being frightened *(“reshaping’… has helped me to be not frightened”)*. She underlines flexibility as a particular constant of her entire professional development, due to her close relationship with many Mathematics Education researchers. Among the several professional development courses she attended, the training on CT seems to have had a particularly important impact leading to the reflection and renewal of her own teaching strategies.

Sophia: My first reaction [to some Chinese teaching methodologies] was one of amazement and curiosity […] I immediately thought I could* "copy" * certain strategies and methodologies, hoping that they would have the same effect in my classes. Only by continuing my training I understood that this could not work; a *"transposition"* was necessary from the Chinese school to the Italian one, so that these ways of working had an impact on my children as well. I immediately interpreted the concept of variation, for example, as a* "trick"* that would have allowed all my children (even the most fragile) to solve some mathematical problems. Only later I realized that ‘variation’ is a much more extended concept: it is the ability to make the arithmetic concept *"fluid"*, able to* "flow" * in one direction or another, and in doing so allowing children to manipulate numbers and mathematical meanings in many ways and from many different points of view. […] It would not have been possible to give meaning and a proper didactic intentionality without this study on the basis of Italian and Chinese teaching. My way of teaching has changed profoundly, as has the meaning I gave to the things I did and do in class with the children. […] The results obtained both in motivation and in the profit of my students convinced me to continue on this way, renewing myself and renewing my way of teaching more and more.

We believe that this part of Sophia's interview is particularly important because it shows how her words contain several indications of a change in the *Symbolic* triggered by teacher training processes. For example, in the previous excerpt we can see an expansion and a modification of Sophia’s understanding of the concept of variation in arithmetic, due to her training on problems with variation from the Chinese culture. The concept of ′*variation',* proper of the Chinese culture, certainly is not part of Sophia's *Symbolic*, since she was born and raised in another culture: nevertheless, her training in CT allowed her firstly to realize this 'lack' of that concept in her *Symbolic (“immediately interpreted the concept of variation as a ‘rick’”[…] Only later I realized that 'variation' is a much more extended concept"*), and subsequently to take over this concept through a reinterpretation of it on the basis of elements of her personal *Symbolic*. Moreover, this change – since it develops within a CT process that raises a reflection on one's own cultural ″un-thoughts″ – becomes radical and significant enough to affect the teacher's own *Imaginary.* For example, in the very conclusion of this passage, the teacher expressly talks about *“renewing myself and renewing my way of teaching more and more”*.

The importance of being flexible and available to question one own's teaching methods according to Sophia is also emphasized when she talks about the skills and the abilities she thinks teachers need to overcome the current situation.

Sophia: The teacher who wants... who sees him/herself in a certain role, who still wants to live his/her profession in a certain way, that kind of teacher will find him/herself asking questions, growing, updating, confronting his/her colleagues, looking for elements for improvement in these situations; who doesn't - but also for his/her own personal nature, ok? - who does not have this type of approach can get out of an emergency of this kind as s/he was a month and a half ago.

From Sofia's words an interesting element emerges in view of re-thinking training courses. If we notice the repeated use of the reflexive personal pronoun *(“who sees *him/herself* in a certain role, who still wants to live *his/her profession* in a certain way, that kind of teacher will find *him/herself* asking questions […] for *his/her own personal nature*”)* we can identify a particular attention to the *Imaginary* order. We think this refers to alienation processes to which the teacher’s mirror image undergoes. When reality triggers personal crisis, flexibility and “re-shaping” become fundamental to maintain a process of continuous rebalancing between *Imaginary*-*Symbolic* orders and reality, a process described by Sophia with the words *“asking questions, growing, updating, confronting his/her colleagues, looking for elements for improvement in these situations”*. If the teacher is not open to a continuous process of own redetermination, alienation produces a sort of inability to rebalance anything: at this point, the teacher leans completely on the *Symbolic* order referring to teaching in presence, ignoring reality and refusing to change own teaching methods (*“can get out of an emergency of this type as s/he was a month and a half ago”)*. We can see also a third possibility that Sophia does not mention: when the *Symbolic* order of the teacher is less consolidated, and so it is difficult to lean completely on it, the teacher can read the misalignment between the *Imaginary*-*Symbolic* orders and reality as impossible to adjust, feeling a sense of powerlessness, and then s/he can only wait for the crisis situation to end.

## Discussion and concluding remarks

In the attempt to explore the needs and suggestions for teacher training emerging from the crisis situation experienced by mathematics teachers in COVID-19 time, in this paper, we used Lacan's psychoanalytical lens and presented a case study of an Italian expert teacher, Sophia. We interviewed her twice one month apart, during the lockdown period and an exclusive school organization in DE mode.

Our data show how, in this context of crisis, the *alienation* towards the reality – school lockdown – makes Sophia’s self-image *out of joint* with respect to the current school structure. Faced with this situation of misalignment, she then tries to recompose an equilibrium starting from a crucial element: the interaction with her pupils. We see this as the key element of Sophia’s image of herself and her role (*Imaginary* order), which allows her to begin to search for suggestions to design and carry out lessons in DE without losing too much coherence with her *Symbolic* order. We think that her searching efforts show, in return, both an imbalance between her *Imaginary*-*Symbolic* orders and reality, and an attempt to realign them. These orders – that are based on perspectives on education that are far from the constraints of DE – were actually built through Sophia’s immersion in a culturally and politically determined context (Italian school) and through her participation to professional development courses.

In particular, Sophia’s way of coping with this misalignment consists in a search for a new way of enacting a selection of the elements at the basis of her *Imaginary* and *Symbolic* orders in the current DE context. Starting from her desire to restore her relationship with her students, she looked for solutions that would allow her to stay in contact with them, without losing the focus on mathematical contents. Even in front of a crisis of her "semiotic mediation approach” to Mathematics teaching and the loss of the “semiotic dimension” of her teaching – for example use of teamwork on manipulatives – she attempted to design and carry out lessons simulating the dynamics she had in her previous way of teaching. These solutions allow Sophia to temporarily realize lessons that are in line with some of the fundamental aspects of her view of teaching. In this perspective, Sophia’s subjectivity is altered mostly in the way she enacts her fundamental beliefs on teaching rather than in the view of her role as a teacher and her view of what school should be.

We think it is significant that Sophia identifies some strong thematic issues in DE, such as: the human relationship with students, available time, and issues concerning lesson planning. This last point becomes the cornerstone of a didactic activity recontextualized in DE and aimed at rebalancing the initial situation of imbalance. In order to cope with the strict time constraints of DE, she exploits her solid training on CT of Lesson Study (*Symbolic* order) to enhance her lesson planning analysis: she uses it to design lessons in greater detail, and to carefully establish the timing of lesson crucial phases. Sophia is aware that it is not possible to maintain in DE teaching methods used in presence, but it is necessary to be flexible, in order to “reshape” one's own image as a teacher, even in a completely changed real context.

Now, we can address a series of questions that indicate several directions of research development. We will try to trace out some possible trajectories, which obviously do not to saturate all the possibilities, but that emphasize some possible links that seem essential to us.

In the face of the current global crisis situation, there seems to be a lack of studies providing useful indications on how to design training courses to develop teachers’ skills to cope with difficult situations, and to develop resilience attitudes. If we observe the current proliferation of training courses on platforms, tools, materials, etc., on DE, we believe that Sophia’s case shows that technological skills and knowledge, though obviously indispensable, alone are not enough for teachers to be able to react, if there is no training on other aspects as well. In particular, Sophia’s words raised issues concerning: how to conciliate a view of teaching based on a “semiotic mediation approach” (materials, manipulatives, mathematical discussions) with DE, how to manage lesson planning and task design in relation with short lesson time, how to “reshape” one’s own way of teaching in relation to the constraints lockdown imposed. Sophia’s case suggests a key skill needed to face crisis situations: flexibility – i.e., the capability to adapt to current contextual conditions, exploiting one's own background and professional development. We think more research is needed to investigate if it is possible to develop coping skills such as flexibility through specific training courses. In particular, we hope that further studies will explore whether this reading of the subject's crisis as an imbalance between the *Imaginary*-*Symbolic* orders and reality is useful to further explore relationships between specific skills and the ability to overcome crisis situations.

In relation to these issues we also try to make some proposals for teacher training. According with what we observed before, CT of Lesson Study revealed to be a concrete possibility to train teachers in managing imbalance. Indeed, it concerns an approach to an a priori lesson analysis, and the development of teachers’ critical thinking – aspects that are transversal to the specific content to be taught (Bartolini Bussi et al., 2017). Moreover, as we have seen in the development of pre-service teachers faced with Lesson Study (Bartolini Bussi & Funghi, 2019), experiencing Lesson Study can alter culturally determined beliefs regarding what it takes to carry out mathematics lessons or how to design them. Through Brown’s approach on subjectivity, we can read the CT of Lesson Study experience as an opportunity for the teacher to live a small imbalance at the *Symbolic* order, intentionally designed by teacher educators, and to exploit it, in order to reflect on one’s own educational intentionality. We think that professional development courses focused on Lesson Study can create disequilibrium situations and give teachers the opportunity to deal with them.

More in general, cultural distance generates a situation of imbalance which forces the possibility of didactic deconstruction processes (Mellone et al., 2020)—an imbalance that we situate in the *Symbolic* order. We note that Sophia undertook this same process of deconstruction in a “restricted” form: namely, the deconstruction of a didactic practice defined in another culture (Chinese Lesson Study) was aimed to adapt it not to her culture as a whole, but to contingent, local needs. This aspect addresses reflections to be explored in further research on the potential and limits of CT: if CT in its original construct seems to generate imbalance in the *Symbolic* order, Sophia’s choice makes us wonder about the possibility to study CT in a wider perspective, in relation to imbalances situated not only in the *Symbolic* order, but also between the *Imaginary*- *Symbolic* orders and reality*.*

According to these considerations and Sophia’s suggestions, a possible proposal for a professional development course design could focus on teachers’ detailed lesson design (for example a priori Lesson Study analysis). Taking more general perspective, we believe that a teacher training course aiming to promote changes in teachers should take into account a subjectivity dynamic structure, for instance, by creating situations of strong imbalance to provoke a search for re-balancing – as we have seen in the case of professional development courses about Chinese problems with variations for Sophia. To this aim it is necessary first of all to focus on the *Imaginary* and *Symbolic* characterizing trained teachers. With the CT teacher educators should try to propose professional development courses that are able to affect the *Symbolic* in order to produce a strong influence also on teacher's *Imaginary*. Finally, we would like to further investigate to what extent the teacher educator can push imbalance in the *Symbolic*, without being too demotivating.

## Appendix

**Table:**

Description	My expectations, or considerations	Times
Short initial greetings and recap of needed materials: 24 maccheroni, and 3 plates. Pencil and notebook		5 min
We take the notebook and we write: “Let’s prepare the plates”. I show pupils the PowerPoint with the task and I dictate the text		5 min
Task: ***“Take 24 maccheroni and put it on the table*** Today at lunch will be only 2 persons, let’s prepare the plates for them. Since we want them to have the same number of maccheroni, **how many maccheroni should I give to each of them?”**	There will be children distributing 1 maccherone at a time and other distributing groups of more than 1 maccherone (for example 2, 5 or 10 at a time)	5 min
Now take the notebook and write down how you have completed the task. Describe in detail everything you had to do to discover how many maccheroni you should give to each of your guests. Tomorrow you have to send me a photo of your notebook	Pupils write down on their notebook	5/10 min
**“How did you make it?”** I listen to pupils as they finish to write; I ask to everyone to raise their hands when they have done	If all pupils distributed 1 maccherone at a time, I ask “Could we accomplish the same task more quickly?”	10 min
**“What have we done with our maccheroni?”**	I expect for words as “distribute” or “divide” to come out in pupils’ explanations. In the unlikely event that pupils do not use these words, I will tell them that another child of another class told me that s/he divided/distributed, and I will ask to my pupils what do they think about it	5 min
We take the notebook and write: *“Every person receives 12 maccheroni because we have divided/distributed maccheroni to each of them”* In “math writing” 24:2 = 12 because 12 × 2 = 24		
Greetings		2 min
**In case we have extra time**		
Task: “Now we have 3 guests at lunch. Add a plate to those you have on your table Put all maccheroni on the table again, and try to divide/distribute to each guest the same number of maccheroni”	There will be pupils distributing maccheroni in groups of more than 1 maccheroni. Perhaps, someone is already able to see that 24:3 = 8 because 3 × 8 = 24 ***Anyway, I will emphasize the possibility to gradually “empty” the maccheroni bunch on the table***	

## Footnotes

1 Here and in the sequel, we prefer to use the Italian word *“educatore”* instead of the English word "educator", to underline its different meaning in the Italian cultural context.
